# The change of psychosocial stress factors in families with infants and toddlers during the COVID-19 pandemic. A longitudinal perspective on the CoronabaBY study from Germany

**DOI:** 10.3389/fped.2024.1354089

**Published:** 2024-03-18

**Authors:** Catherine Buechel, Anna Friedmann, Stefan Eber, Uta Behrends, Volker Mall, Ina Nehring

**Affiliations:** ^1^ Chair of Social Pediatrics, TUM School of Medicine, Technical University of Munich, Munich, Germany; ^2^ Professional Association of Pediatricians in Bavaria (BVKJ) and PaedNetz Bayern, Munich, Germany; ^3^ Children’s Hospital, School of Medicine, Technical University Munich, Munich, Germany

**Keywords:** parent psychosocial functioning, infant mental health, COVID-19 pandemic, early life adversity, parenting stress, depression, anxiety

## Abstract

**Background:**

Over nearly three years, the COVID-19 pandemic has had a lasting impact on people's lives and mental health worldwide with its far-reaching restrictions and concerns about infections and other personal consequences. Families were particularly affected and showed increased stress and psychological problems. Long-term effects cannot be ruled out. So far, data on young families are sparse. The present longitudinal analysis (*n* = 932) of the CoronabaBY study investigated the development of parenting stress, parental affective symptoms, and child's mental health in young families with children aged 0–3 years in Germany as well as potential influencing factors.

**Methods:**

The observational study includes two measurement points over the course of the pandemic (baseline and follow-up). Data was collected by app using standardized questionnaires.

**Results:**

*N* = 932 participants, mainly mothers (94.7%) born in Germany (93.1%) with higher education (61.3% with at least high school diploma) and a comfortable financial situation participated in the longitudinal study. Children were on average 14.7 months old at baseline (SD: 12, range: 1–39 months). While the proportion of parents who perceived the pandemic as stressful decreased significantly from baseline (60%) to follow-up (52.3%), the proportion with parenting stress increased significantly (from 40.1% to 45.4%). Both parental and child mental health problems remained constant over time, with infants crying/feeding/sleeping problems ranging above pre-pandemic comparative data. Most predictive for high parenting stress at follow-up was high parenting stress at baseline. This was also true for parental affective symptoms (depression/anxiety) and child mental health problems.

**Conclusions:**

Despite faded pandemic restrictions, parents remained burdened. Support services do not appear to have been sufficient to help families out of their stressful situation. Our results indicate a need for action regarding low-threshold services that effectively reach affected families.

**Trial registration:**

The study was pre-registered in OSF (https://osf.io/search/?q=tksh5&page=1).

## Introduction

For nearly three years, the COVID-19 pandemic has preoccupied the world, leading to a prolonged state of emergency with far-reaching restrictions and impact on everyone's live (1, 2). Families were particularly affected by additional childcare responsibilities due to the closures of day care centers/schools (3), disruptions in daily routines and limited access to family support services (4). They showed a high level of multiple stress factors and burden due to restriction measures (3, 5–8) which was seen especially in families with very young children (3, 7, 8). Fear and worries as well as social isolation are supposed to have caused acute states of stress at the onset of the pandemic. Long-term, however, a chronification of stressors is likely (8). Even short-term relaxations of high Covid-incidence rates and fading of restriction measures in Germany did not reduce psychosocial burdens in families (8). In accordance with the assumption that chronic stress can have lasting impact on mental health (9, 10), experts predicted a wave of mental illness following the wave of infection (11).

Various studies showed an increase in psychosocial stress factors among parents and children, including increases in parenting stress (12–15), parental mental health symptoms such as depression and anxiety (16–22), and child's psychological problems (13, 20, 22–32). However, most of these studies only reported on the first pandemic year whereas longitudinal studies mainly compared their results to pre-pandemic surveys (33). Moreover, children's age was at least school age (e.g., (23), investigations on early childhood are sparse (33).

Regarding the importance of early childhood for a healthy development, long-term data on infants, toddlers and their parents during the pandemic from a longitudinal perspective are needed to further assess mental health in young families (34–36). Early psychosocial stress in childhood can have a potentially harmful influence on a child's mental health (37–39). In addition, families with young children can be considered as a specific risk group (3) as infants and toddlers are still highly vulnerable to external influences and exclusively dependent on their parents’ involvement in care and emotional availability (40, 41). Parenting-related exhaustion was notably higher during lockdown the younger the children were (42) and well-being significantly decreased for parents with young children in times of COVID-19 (3). Understanding young families’ psychosocial needs is fundamental for developing and addressing adequate support services.

The CoronabaBY study investigated psychosocial stress factors of families with children aged 0–3 years in Germany (7, 8). While comparing three samples from three pandemic waves in a cross-sectional observation (February 2021–March 2022) (8) the extent of the perceived pandemic burden followed the waves and their attending restrictions. Parenting stress and crying/sleeping problems of infants, however, constantly increased and were higher in families who were examined later (October 2021–March 2022) than earlier during the pandemic (February–June 2021). At the same time, parental depression and anxiety symptoms were elevated in all three pandemic phases—independent of current infection rates or restrictions. In summary, psychosocial stress factors were highly pronounced regardless of the degree of pandemic restrictions/relaxation of measures (8). However, these findings are based on a comparison of cross-sectional data. Although the participating families in the three waves showed similar sociodemographic characteristics, the results do not provide intra-individual observations of the same sample. Thus, to detect the development of psychosocial stress factors as well as underlying predictors within the sample over the course of the pandemic, we conducted a longitudinal analysis.

The present evaluation aims to extend the previous study findings and to show intraindividual changes and trends during the pandemic, considering two measurement points (baseline and follow-up). This leads to the following research questions:

1. How did the experienced psychosocial stress factors (parenting stress, parental affective symptoms, child mental health symptoms, and perceived pandemic burden) change in the sample over the course of the pandemic (baseline to follow-up)?
2. Which pandemic-related (e.g., increased family conflicts) or sociodemographic factors (e.g., financial situation, education level) influenced the psychosocial stress factors perceived in the families (parenting stress, parental affective symptoms, child mental health symptoms) over the course of the pandemic, i.e., in the follow-up?

## Materials and methods

### Study design

The CoronabaBY study investigated intermediate and long-term psychosocial stress during the COVID-19 pandemic (“Corona”) in families with infants and toddlers (“baby”) in Bavaria (Southern Germany) (“BY”). Data has been collected continuously from the 1st of February 2021 until the 2nd of November 2022. Data was evaluated longitudinally, i.e., at two measurement points (baseline and follow-up). The study protocol was approved by the Ethics Committee of the Technical University of Munich (vote no. 322/20 S) and pre-registered in OSF (https://osf.io/search/?q=tksh5&page=1).

### Participants

All participants (parents with children up to three years) were recruited and surveyed via the smartphone app “Meine pädiatrische Praxis” (“My pediatrician”) (www.monks-aerzte-im-netz.de), which is a well-established communication tool connecting parents with their pediatrician [for detailed recruitment information, see (7]). Invitations to the study were sent out together with invitation to the next early childhood check-up (“U-Untersuchung”). Therefore, measurement time depended on time of early childhood checkup. At baseline, the first checkup considered was “U4” (child aged around 3–4 months) and the last was “U7a” (child aged around 34–36 months), thus the ages of the children ranged around 3 months and 3 years. Corresponding reminders via app acted as invitations to the in-app-baseline respectively -follow-up-surveys. Due to the varying time intervals between the different checkups, there were individual time intervals between baseline and follow-up for the families. All children up to checkup “U6” (about one year) were classified as “infants” in this study; children from “U7” on (about two years) were considered “toddlers”.

**Figure 1:**
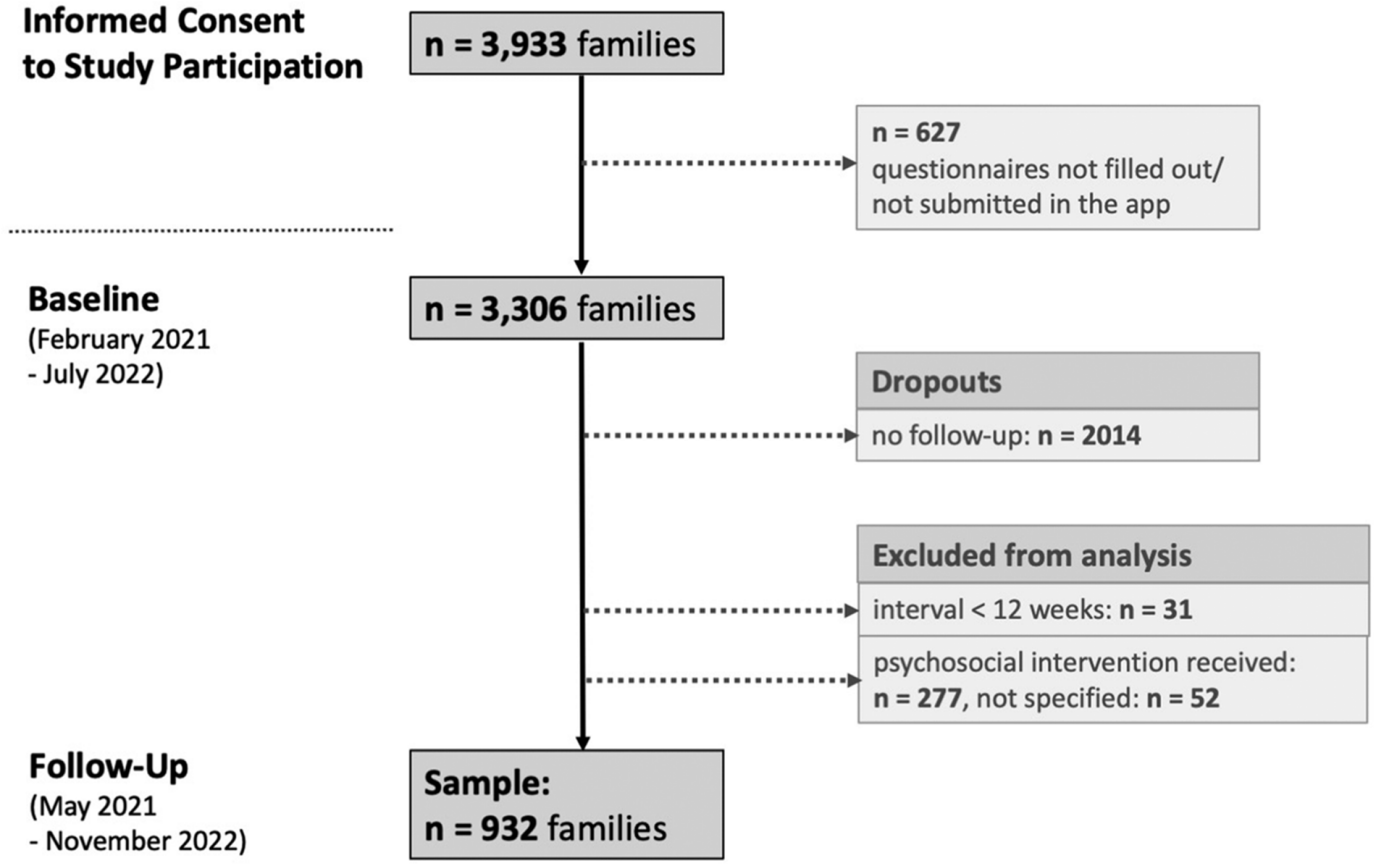
Numbers of participating families at baseline and follow-up of the CoronabaBY study.

Informed consent was given by nearly 4,000 parents, a total of 3,306 finally attended and completed the in-app-study-questionnaire at baseline. A remaining number of 932 parents (28%) could be included in the follow-up evaluation (see Figure 1).

### Measures

All data was collected by standardized questionnaires via app. Participants were asked about general sociodemographic characteristics, perceived pandemic burden, parenting stress, and parent and child mental health outcomes.

#### Pandemic-related restrictions and perceived pandemic burden

Ten questions were asked about specific restrictions and perceived burdens related to the pandemic (e.g., “During the strictest pandemic measures, how restricted did you feel about social contacts?”). The perceived “pandemic burden” for parents and children was derived from the 5-point-answer (from 1 = *not at all stressful* to 5 = *very stressful*) to the global question: “Taken together, what do you think: How stressful is/was the COVID-19 pandemic for you (please think of measures like social restrictions but also your personal experiences, related worries,…)?” and “Taken together, what do you think: How stressful is/was the COVID-19 pandemic for your child?”, respectively. The study team developed the questions due to the lack of validated instruments at this point in the pandemic. Previous publications on the CoronabaBY study could show that these questions on pandemic burden represent the pandemic in a comprehensible way (perceived stress due to the pandemic follows the degree of restrictions) and correlate significantly with each other as well as with other psychosocial stress factors (e.g., Parenting Stress Index, PSI) (7, 8).

#### Parenting stress

To assess parenting stress, we applied the parent domain of the German Version of the “Parenting Stress Index (PSI)” *[*“*Eltern-Belastungs-Inventar*” *EBI*; (43]). High scores indicated limited parental resources for upbringing and care for the child. The parent domain includes the following subscales: “health” (parental health impairment as a cause or a result of parenting stress), “isolation” (lacking integration in social networks), “role restriction” (perceived limitations as a result of being parent), “parental competence” (parental doubt about their abilities to manage upbringing and care for their child), “attachment” (emotional relation of parent on the child), “depression” (limited emotional availability within the parent-child-relationship) and “spouse related stress” (as a result of being a parent). Answers were given on a 5-point Likert scale ranging from 1 = *strongly agree* to 5 = *strongly disagree,* resulting in a possible score range of 28–140. The three cut-off categories for each subscale and the whole parent domain were “*not stressed*” (T-value < 60), “*stressed*” (T-value = 60–69), and “*strongly stressed*” (T-value ≥ 70) (43). Internal consistency of the parent domain has been proven to be good (*α* = .93), and retest reliability after one year is *r* = .87. Correlations with stress indicators and related constructs have resulted in the assumption of test validity (43, 44).

#### Parental depression and anxiety symptoms

Current parental depression and anxiety symptoms were assessed with the State-Trait-Anxiety-Depression Inventory [STADI; (45]). The questionnaire, including four subscales (“emotionality”, “worry”, “anhedonia”, and “dysthymia”), was answered on a 4-point scale ranging from 1 = *not at all* to 4 = *very much*, resulting in a possible score range of 20 to 80. Based on age- and sex-dependent standardized cut-off T-values, each domain (“depression”, “anxiety”, “total”) was defined by symptoms to be “*far below average*” (T-value < 30)*, “below average’* (T-value = 30–39)*,* “*average*” (T-value = 40–60)*,* “*above average*” (T-value = 61–70), or “*far above average*” (T-value > 70). Internal consistency of the global State-Scales (*α* = .92), the State-Depression-Scale (*α* = .87), and the State-Anxiety-Scale (*α* = .90) have been proven to be good. Validity can be assumed based on comparison with other test procedures (46).

#### Infants’ crying, sleeping and feeding problems and toddlers’ emotional and behavioral problems

For infants (until checkup U6, about one year), the two subscales “crying/whining/sleeping” and “feeding” of the Questionnaire for Crying, Feeding and Sleeping [CFS; (47]) were applied. Parents answered 38 questions on behaviors in their infants. Answers were given on 4-point scales, and mean values were calculated (ranging from 1 to 4). According to validated cut-off values, the dichotomous outcome *noticeable problems* and *no problems* were calculated for the domains “crying/whining/sleeping” (cut-off value: 1.84, sensitivity: 87%, specificity: 92%) and “feeding” (cut-off value: 1.27, sensitivity: 57%, specificity: 77%). The validity of the questionnaire has been secured by the proof of high internal consistencies of the scales and by correlations with behavior diaries and clinical diagnoses (47).

For toddlers (from checkup U7 on, about two years), the Strengths and Difficulties Questionnaire [SDQ, short form of the German Version; (48]) was used to examine emotional and behavioral problems. Parents were asked to classify the individual characteristics to be *not true*, *somewhat true,* or *certainly true* for their child in four domains (“emotional symptoms”, “conduct problems”, “hyperactivity/inattention”, and “peer relationship problems”), resulting in a score range of 0 to 40 points. Cut-off values indicated child behavior to be “*no problems*” (0–13 points), “*borderline*” (14–16 points), or “*noticeable problems*” (17–40 points). Internal consistency has been shown to range between *α* = .73 and *α* = .86. By comparison with other corresponding scales (e.g., Child Behavior Checklist), the instrument's validity can be assumed (49, 50).

### Statistical analyses

The present longitudinal study was based on data collected between February 2021 and November 2022. If the two measurement points were closer than 12 weeks (*n* = 31) the family was excluded from the analyses.

Statistical differences between the sociodemographic and psychosocial characteristics of the follow-up participants (= sample) vs. the dropouts were detected using the Chi-square test for categorical and *T*-test for continuous variables.

To answer the first research question, Chi-Square Tests and where appropriate *T*-Tests were calculated to detect potential differences in the proportions of the addressed psychosocial stress factors for baseline and follow-up. If appropriate, the outcome variables were dichotomized: Perceived pandemic burden was dichotomized into stressful/ very stressful (points 4 and 5 on a 5-point Likert-scale) compared to less stressful (points 1–3). Parenting stress (EBI) was classified into stressed/strongly stressed vs. not stressed. Parental mental health problems (STADI) were dichotomized into above average/ far above average vs. average/ below average/ far below average, and toddler’s emotional and behavioral problems (SDQ) into borderline/ noticeable problems vs. no problems. A cut-off variable was available for the CFS subscore crying/whining/sleeping, dividing symptoms into noticeable problems vs. no problems. To find out to what extent a stress factor at baseline determines itself at follow-up, logistic regression models were conducted. The outcomes (EBI, STADI, CFS subscore crying/whining/sleeping, SDQ) were dichotomized as described above. We adjusted for those variables that had a significant effect in the multiple linear regression models (see Table 1). Conditions for calculating the logistic regression models (i.e., no multicollinearity between predictor variables) were checked.

**Table 1:** Multiple linear regression models for follow-up EBI, STADI (mothers), crying/whining/sleeping (CWS) and SDQ score.

Outcomes	EBI total score T value (FU)^a^				STADI total score (mothers) (FU)^b^				CWS sub score (FU)^c^				SDQ total score (FU)^d^			
	*B*	*SE B*	*ß*	*R²*	*B*	*SE B*	*ß*	*R²*	*B*	*SE B*	*ß*	*R²*	*B*	*SE B*	*ß*	*R²*
	* *			.*600*	* *			*.460*	* *			.*348*	* *			*.439*
Predictors																
EBI Total Score T value (baseline)	0.669	0.024	.675**						0.001	0.002	0.015		0.040	0.024	0.078	* *
STADI total score mothers (baseline)					0.475	0.030	.473**									* *
CWS sub score (baseline)									0.503	0.056	.530**					* *
SDQ total score (baseline)													0.539	0.046	.536**	* *
Interval (weeks) btw. baseline & FU	0.053	0.016	.084**		0.038	0.023	.051		0.002	0.003	.078		0.028	0.020	.057	* *
Perceived pandemic burden parent FU	0.876	0.250	.091**		0.287	0.362	.025		−0.015	0.022	−.044		0.037	0.223	.008	* *
Social contacts FU	0.372	0.257	.043		0.442	0.369	.043		0.000	0.021	−.001		0.687	0.249	.151*	* *
Family support services FU	−0.271	0.227	−.036		−0.100	0.327	−.011		−0.002	0.017	−.008		0.022	0.229	.005	* *
Increased Family Conflicts FU	1.060	0.232	.121**		2.584	0.328	.250**		0.061	0.020	.200*		−0.005	0.216	−.001	* *
Changes in child-care situation FU	0.173	0.207	.022		0.087	0.295	.009		−0.004	0.019	−.013		−0.066	0.186	−.017	* *
Fear of COVID-infection FU	0.227	0.183	.031		0.803	0.262	.092*		0.013	0.015	.050		−0.113	.174	−.030	* *
Financ. burden due to pandemic FU^e^	−0.020	0.543	−.001		1.020	0.780	.038		−.015	0.048	−.019		0.595	0.499	.052	* *
Age child FU	−0.005	0.019	−.007		0.028	0.028	.031		−0.002	.014	−.016		0.110	0.036	.128*	* *
Age parent	−0.018	0.049	−.008		−0.010	0.079	−.003		0.005	0.005	.058		−0.056	0.041	−.055	* *
Single parent	−0.302	0.897	−.008		0.732	1.259	.016		−0.062	0.082	−.041		−0.441	0.887	−.020	* *
Chronic illness/disability child^e^	0.830	0.739	.025		0.584	1.041	.015		−0.021	0.078	−.014		0.652	0.602	.044	* *
Parental education^e^	0.377	0.460	.019		−0.693	0.656	−.029		−0.040	0.039	−.057		−0.515	0.424	−.051	* *
Financial situation^e^	0.453	0.471	.023		0.107	0.676	.005		0.018	0.039	.026		−0.356	0.441	−.036	* *

^a^ *N* = 844.

^b^ *N* = 787.

^c^ *N* = 264.

^d^ *N* = 375.

^e^ Dichotomized.

Significance indicated by *p ≤ .05, **p ≤ .001. FU, follow-up.

Regarding the second research question, we addressed which factors might have contributed to the surveyed psychosocial stress factors at follow-up. To check the stability of psychosocial stress factors over time, we included the corresponding factors at baseline, as well as pandemic-related factors and sociodemographic factors as potential predictors. We explored if and to what extent these factors predicted parenting stress (EBI total score, T-value), maternal depression and anxiety symptoms (STADI total score, T-value), infants’ crying/whining/sleeping problems (subscore of CFS crying/whining/sleeping scale), and toddlers’ emotional and behavioral problems (SDQ total score) at follow up. Four multiple linear regression models were calculated. The individual predictors considered were chosen on the basis of previous evaluations of the CoronabaBY study and were in detail: the respective psychosocial stress factors at baseline (EBI total score, STADI total score, CFS crying/whining/sleeping subscore, SDQ total score), pandemic related variables (baseline-follow-up-interval in weeks, perceived pandemic burden at follow up, restricted parental social contacts at follow up, restricted family support services at follow up, increased family conflicts at follow up, changes in childcare due to pandemic at follow up, worries about infection at follow up, financial burden due to pandemic at follow up) and sociodemographic variables (child age at follow up, parents age, single parent status, chronical illness/disability of the child, parental education status, parental financial status before the pandemic). The formation of the models resulted in the calculation of beta weights and their *p*-values for corresponding predictor variables. Conditions for calculating the multiple linear regression models—including linear association between dependent and independent variables, homoscedasticity, normally and independently distributed residuals, no multicollinearity between predictor variables—were checked.

For the linear regression models, four independent variables had to be dichotomized since the scale level was not interval scaled. Consequently, education status was dichotomized into *high* (university degree and high school diploma) and *low* (secondary and lower secondary school diploma). Financial status was also dichotomized into *high* (“large expenses possible” and “bigger additional expenses possible”) and *low* (“smaller additional expenses possible”, “little scope for additional expenses”, “additional expenses not possible*’*). Accordingly, the financial burden due to the pandemic was dichotomized (yes: small, medium, or substantial financial burden vs. no financial burden due to the pandemic). Chronic illness or disability of the child was defined as any chronic illness (also allergy, hyperactivity) and/or disability.

Since submission of questionnaires was only possible when all items were completed, there were only a few missing values because of obvious misreporting of parental age.

All described results were based on an alpha level of 5%. Analyses were performed in IBM SPSS Statistics Version 29.0.

## Results

### Sample characteristics

In total, we examined 932 parent-child dyads with full information at baseline and follow-up (“sample”). *N* = 2014 participants did not submit the follow-up questionnaire (“dropouts”). Of the surveyed parents, 94.7% (*n* = 883) were mothers with a mean age of 33.7 years (SD: 4.7), 4.6% were fathers (mean age: 34.9 years, SD: 5.5), and 0.6% were grandparents. Children were on average 14.7 months old at baseline (SD: 12, range: 1–39 months). They were divided into “infants” (*n* = 518) with a mean age of 5.1 months (SD: 3.4) and “toddlers” (*n* = 414) with a mean age of 26.8 months (SD: 6.6). On average, participants completed the follow-up questionnaires around 40 weeks after baseline (*M *= 39.55, *SD *= 15.94).

### Sample vs. Dropouts

Sample and Dropouts differed significantly concerning sociodemographic factors: in the sample there were significantly more often mothers, born in Germany, with German mother tongue and higher financial status, less often single parents and less often parents of children with chronic illness and/or disability (see Table 2).

**Table 2:** Sample characteristics.

	Sample	Drop-outs	*p*
	% (*n*)		
Parents			
Mothers^a^	94.7 (883)	91.8 (1,848)	0.004
Born in Germany^a^	93.1 (868)	90.5 (1,822)	0.017
Mother tongue German^a^	94.4 (880)	91.3 (1,839)	0.003
Level of education			0.485
University degree	42.6 (397)	41.1 (827)	
High school diploma	18.7 (174)	17.7 (356)	
Secondary school diploma	28.4 (265)	29.5 (594)	
Lower sec. school diploma	8.3 (77)	8.8 (178)	
Other	1.9 (18)	2.8 (57)	
High financial status^b^	60.3 (528)	56.1 (1,041)	0.038
Single parent status^a^	6.2 (58)	8.4 (170)	0.038
Vaccinated, respondent at FU	92 (856)		
Vaccinated, partner at FU	92 (856)		
Children			
M_age_ infants	5.1 months, SD = 3.4 (518)	6.2 months, SD = 4.3 (865)	<0.001
M_age_ toddlers	26.8 months, SD = 6.6 (414)	27.0 months, SD = 7.2 (1,148)	0.479
Boys	51.1 (476)	53.0 (1,068)	0.341
Chronic illness and/or disability^a^	6.6 (61)	9.3 (185)	0.015

^a^ Outcomes dichotomized.

^b^ Outcome (scale) dichotomized into: high (very large/large additional purchases possible) vs. low (small/very small/no additional purchases possible). FU = follow-up.

At baseline, 48% of the dropouts experienced parenting stress which is significantly more often than in the sample (40.1% with parenting stress at baseline). Dropouts showed significantly more often symptoms of depression and anxiety (29.8%) compared to the sample (20.6%). At baseline, significantly more dropouts reported a high pandemic burden for their child (36.4%) than participants of the sample did (30.3%).

### Perceived pandemic burden and pandemic-related restrictions

**Figure 2:**
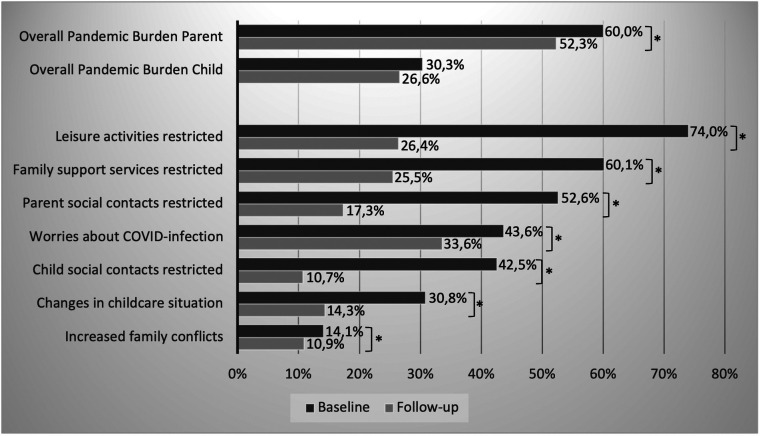
Percentage of parents/children with noticeable values in pandemic-related burdens at baseline and follow-up (*indicates a sign. difference with *p* ≤ .05).

At baseline, 60% of the parents of the sample perceived the pandemic as stressful or very stressful. This proportion decreased significantly to 50% at follow-up. Almost one-third of the parents rated their children's pandemic burden as high or very high at baseline, which did not significantly decrease until follow-up. A significant reduction of individually perceived pandemic-related restrictions was detected (see Figure 2).

### Parenting stress and parental mental health

**Figure 3:**
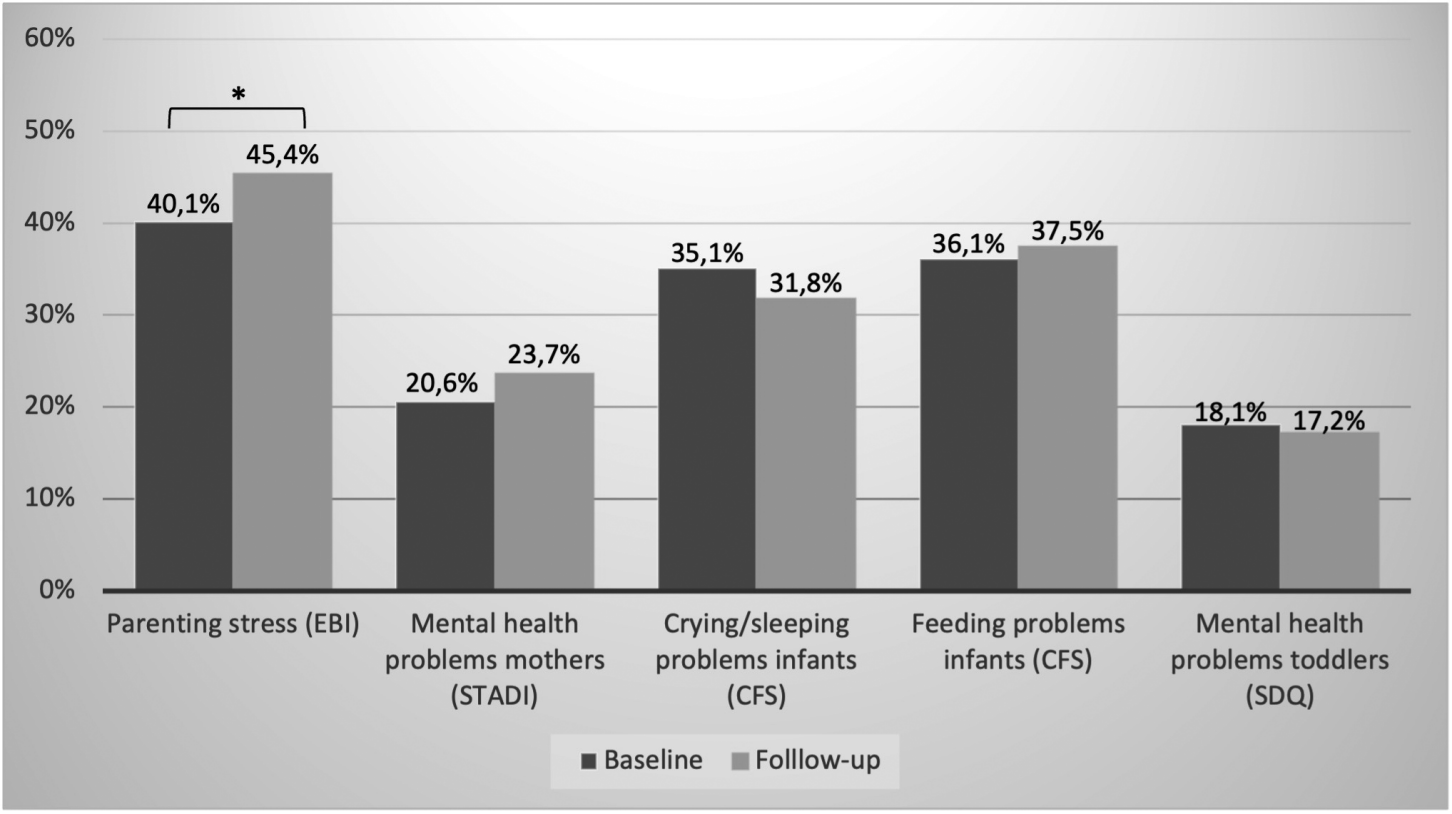
Percentage of parents/children with noticeable values in psychosocial stress factors at baseline and follow-up (*indicates a sign. difference with *p* ≤ .05).

High or very high parenting stress was present in 40.1% of the parents at baseline and increased significantly to 45.4% at follow-up (see Figure 3). This difference is also evident in the mean values: the comparison of the mean EBI total T-values yielded a mean value of 56.02 at baseline vs. 57.42 at follow-up, *t*(931) = 6.18, *p *< .001, *d* = .20. Of the strongly stressed parents at baseline, 61.4% were still strongly stressed at follow-up, and only 2.4% were not stressed. Over both measurement points, “depression” was the most pronounced parenting stress subscale, followed by “health” and “social isolation”. The proportions were higher for follow-up (64.3% respectively 50.9%, and 48.2%) compared to baseline (61.5% respectively 41.7%, and 44.0%), with the increase only being significant for “health”.

Proportions of maternal anxiety and depression symptoms differed slightly but not significantly between baseline (20.6%) and follow-up (23.7%). Among the parents with conspicuous values far above average (above average) at baseline, still 33.3% (47.2%) were far above average (above average) at follow-up.

### Child mental health (crying, sleeping and feeding, emotional and behavioral problems)

On the CFS's crying/whining/sleeping subscale, there was no significant difference between baseline and follow-up (35.1% vs. 31.8%). The proportion of infants with a feeding problem was similar and did not change significantly between baseline and follow-up (see Figure 3). Of the infants who showed elevated values on the crying/whining/sleeping subscale at baseline, 58.1% were still conspicuous at follow-up. This was similar for those with feeding problems (52.8%).

At baseline, 18.1% of the toddlers showed at least borderline emotional and behavioral problems which remained constant until follow-up (17.2%). Among the toddlers with noticeable problems at baseline, 51.4% were still noticeable at follow-up.

### Risk of perceiving follow-up parenting stress, maternal symptoms of depression and anxiety, infants’ crying/sleeping problems, and toddlers’ emotional and behavioral problems

The adjusted logistic regression models yielded significant Odds Ratios (OR) for all psychosocial outcomes at follow-up (EBI, STADI, CFS crying/whining/sleeping, SDQ) if corresponding symptoms were already noticeable at baseline (ORs ranged from 5.7 to 9.6, Nagelkerkes *R*^2^ from .214 to .421, see Table 3).

**Table 3:** Odds ratios for elevated values of EBI, STADI (mothers), crying/whining/sleeping (CWS) and SDQ-scores at follow-up.

Factor	Outcome	OR (95%CI)	Sig	Nagelkerke *R*^2^
EBI baseline^a^^,^^b^ “stressed/strongly stressed”	EBI follow-up^a^ “stressed/strongly stressed”	9.623 (6.940–13.343)	***	.421
STADI baseline (mothers)^a^^,^^c^ “above average/far above average”	STADI follow-up (mothers)^a^ “above average/far above average”	5.676 (3.842–8.385)	***	.324
CWS baseline^a^^,^^d^ “noticeable problems”	CWS follow-up^a^ “noticeable problems”	6.105 (3.546–10.509)	***	.238
SDQ baseline^a^^,^^e^ “borderline/noticeable problems”	SDQ follow-up^a^ “borderline/noticeable problems”	7.439 (4.161–13.297)	***	.214

^a^ Dichotomized.

^b^ Adjusted for: interval (weeks) between baseline and FU, perceived pandemic burden parent FU, increased family conflicts FU.

^c^ Adjusted for: increased family conflicts FU, fear of COVID infection FU.

^d^ Adjusted for: increased family conflicts FU.

^e^ Adjusted for: social contacts FU, age child FU.

*** *p* < .001. FU, follow-up.

### Influencing factors on follow-up parenting stress, maternal symptoms of depression and anxiety, infants’ crying/sleeping problems, and toddlers’ emotional and behavioral problems

The linear regression model [*R*^2^ = .600, *F*(15, 828) = 82.84, *p* < .001] showed parenting stress at baseline (EBI total score T-value baseline) to have the highest effect size (*β* = .675, *p* < .001) on the follow-up outcome parenting stress (EBI total score T-value follow up), followed by increased family conflicts at follow up (*β* = .121, *p* < .001), pandemic burden at follow up (*β* = .091, *p* < .001) and longer baseline-follow-up-interval (*β* = .084, *p* < .001) (see Table 1). For maternal symptoms of depression and anxiety at follow-up (STADI total score follow-up) [*R*^2^ = .460, *F*(15, 771) = 43.73, *p* < .001], STADI total score at baseline had the highest effect (*β* = .473, *p* < .001), followed by increased family conflicts (*β* = .250, *p* < .001) and fear of COVID-infection (*β* = .092, *p* < .05). Sociodemographic factors did not significantly affect parenting stress and parental mental health symptoms during follow-up (see Table 1).

Looking at infants’ crying/sleeping problems at follow-up (CFS crying/whining/sleeping subscore follow-up), the model [*R*^2 ^= .348, *F*(16, 247) = 8.24, *p* < .001] showed crying/sleeping problems at baseline (CFS crying/whining/sleeping subscore baseline) to have a significant effect (*β* = .530, *p* < .001), as well as increased family conflicts at follow-up (*β* = .200, *p* < .05). Parenting stress at baseline, however, did not significantly affect crying/sleeping problems in infants at follow-up (see Table 1).

For toddlers’ emotional and behavioral problems at follow-up (SDQ total score follow-up) both toddlers’ emotional and behavioral problems at baseline (SDQ total score baseline) (*β* = .536, *p* < .001), the restriction of social contacts at follow up (*β* = .151, *p* < .05) and child's age at follow up (*β* = .128, *p* < .05) had a significant effect in the model [*R*^2^ = .439, *F*(16, 358) = 17.54, p < .001]. Again, parenting stress at baseline did not significantly affect toddlers’ mental health at follow-up.

## Discussion

According to the present results of the German longitudinal CoronabaBY study, parents experienced a significant increase of parenting stress over the course of the pandemic, whereas parental and child affective symptoms remained constant. The percentage of overall perceived pandemic burden and perceived restrictions in parents decreased. Among the factors influencing psychosocial outcomes during follow-up, their counterparts at baseline proved to be most predictive ones. In addition, family conflicts were relevant for higher parenting stress, parental affective symptoms, and infants crying/whining/sleeping problems whereas a higher degree of social contact limitation and increased child age were predictors for toddlers’ emotional and behavioral problems.

Looking at the findings in more detail, significantly less parents perceived the pandemic as highly stressful (“pandemic burden”) at follow-up compared to baseline (52% vs. 60%). This might be due to the fact that most of the follow-up data was collected when restrictions were step by step withdrawn in Germany, and pandemic conditions slightly disappeared. The estimated pandemic burden for children ranged at a much lower level from the beginning and did not change significantly. Most likely, very young children, as considered in the present study, were less directly affected by the pandemic measures.

The proportion of parents who experienced parenting stress at baseline (40.1%) was already high compared to pre-pandemic data [see (7)]. However, although pandemic burden was slightly fading, significantly more parents perceived high parenting stress at follow-up (45.4%). This development was also evident in the comparison of the mean EBI T-values which increased significantly from baseline to follow-up (56.02 vs. 57.42). A previous repetitive cross-sectional analysis of the CoronabaBY study with a comparison of three subsamples also showed an increase in parenting stress over different waves in the 2nd year of the pandemic (8). High levels of parenting stress in the pandemic have already been proven by previous studies, but so far only for the initial phase of COVID-19 [e.g., (12–15]). To our knowledge, there are no comparable studies yet available for the further course or later periods of the pandemic. It is also alarming that two-thirds of the parents constantly showed conspicuously high values in the “depression” subscale of the EBI, i.e., limited emotional availability within the parent-child relationship was indicated. This, in turn, could negatively impact the young child's needs as they are still highly dependent on their caregivers’ external regulation and support for their emotional regulation (41, 51). In addition, the proportion of parents with high values on the EBI-“health” subscale increased significantly. The growing parenting stress in the course of the pandemic, despite a reduced perceived pandemic-related stress, might reflect a stable state of the parental psychosocial symptoms rather than acute reactive stress experiences to relatively short-term changing pandemic restrictions (8, 52). Further, the duration of the pandemic, with no foreseeable end in the meantime, might have led to a perceived prolonged state of emergency and a so-called “pandemic fatigue” (2, 8, 53). Accordingly, already in early 2020, experts described a mental ill crisis that may follow the wave of infections (2, 11). In this study, most follow-up data was collected in 2022 (by 90%) when pandemic restrictions were reduced or removed altogether. This probably explains the lowered perceived pandemic burden, whereas the emergence of new crises (e.g., War in Ukraine, inflation) might have caused a complex stress situation keeping parenting stress on a high level. Proportions of parental depression and anxiety symptoms did not differ significantly between follow-up and baseline (23.7% vs. 20.6%) and still correspond to comparative values from a pre-pandemic German study, where 20.1% of the parents with children under three years of age perceived affective symptoms (54). This is somewhat surprising, since the State-subscale of the STADI was used, which rather depicts short-term affective states. Elevated scores in acute response to the pandemic (or new emerged crises) were expected since other studies have shown higher affective symptoms in parents during COVID-19 [e.g., (55)]. Nevertheless, more than half of the parents with depression and anxiety symptoms at baseline were still conspicuous at follow-up.

Looking at the infants and toddlers, there were no significant changes over time in all psychosocial outcomes measured. This is reassuring and confirms that children under three years of age were probably less directly affected by the pandemic restrictions. However, since infants and toddlers predominantly depend on their parents care and hence might be influenced by their stress (40, 41), a detrimental effect from parents on child symptoms over time, i.e., an increase of child's psychological problems, could be assumed (8). This assumption could not be confirmed in this longitudinal analysis as the noticeable parenting stress at baseline was not a significant predictor of the child's crying/sleeping or emotional and behavioral problems at follow-up. However, the proportion of infants with crying and sleeping or feeding problems remained high until follow-up. While the proportion of toddlers with emotional or behavioral problems is within the normal range (56), the proportion of infants who show problems in regulating themselves exceeds the findings of various studies before the pandemic (57–61), even if not wholly comparable pertaining study design and definition of regulation problems. Presumably, infants have an even more exclusive dependence on the emotional attention of their parents (62). High parenting stress could therefore impact the children's ability to regulate themselves (63–65), although our data did not confirm this effect. With growing age, children have more social contacts outside and go more often to care facilities, which were increasingly facilitated and opened in 2022. This may have also affected the toddlers in our sample which is supported by the finding that the degree of social contact restrictions had an impact on the emotional and behavioral problems of the toddlers in the follow-up. A previous evaluation of the CoronabaBY study on different waves (repetitive cross-sectional comparison) (8) showed significant higher prevalences of problems in crying, whining, and sleeping later during the pandemic (10/21–03/22) compared to earlier (02–06/21), which was not observed in the present longitudinal evaluation. This might be explained by the naturally increased age in the intra-individual follow-up over 3–12 months: Evidence suggests that regulation problems decline with growing age (66), which aligns with the trend in the present findings. Nevertheless, around half of infants and toddlers with noticeable problems at baseline were still conspicuous at the follow-up. According to a German longitudinal study on mental health of children and adolescents (≥7 years) during the three years of the COVID-19 pandemic, affective symptoms (also measured by the SDQ questionnaire) improved in the third year. However, mental health was still lower compared to before the pandemic (23).

To better understand the underlying mechanisms in the longitudinal development of the psychosocial markers, we identified factors influencing them over the course of the pandemic. On both parental (EBI total score, STADI total score) and child outcomes (CFS crying/whining/sleeping subscore, SDQ total score) at follow-up, their respective counterparts at baseline had the most significant influence (small to medium effects), indicating a stable state of these psychosocial burdens over time. The logistic regression models confirmed that being affected at baseline increases the risk for being affected at follow-up by a multiple. Further influencing factors had weak effects. For parenting stress, these were increased family conflicts at follow-up, perceived pandemic burden at follow-up, and a longer time interval between baseline and follow-up. The latter indicates increasing psychosocial stress in parents with the duration of the pandemic and persistent perceptions of related restrictions and burdens. For parental depression and anxiety symptoms at follow-up, increased family conflicts (at follow-up) were also influential, as well as the fear of a COVID-infection (with a weak effect). This is where interventions could come in and show relieving ways of solving and coping with conflict situations. Toddlers’ emotional and behavioral problems increased with a higher degree of social contact limitation and rising child age, which is in line with other studies (67–69). In infants, only increased family conflicts further influenced their symptomatology at follow-up. This is in line with a general population study in Denmark in which regulation problems in early infancy turned out to be the main predictor of late combined regulation problems, i.e., two or more simultaneous problems of feeding, sleeping, or excessive crying (70). Being significantly affected by conflicts in the family also indicates the close sensitivity of the infants to their caregivers. Sociodemographic factors consistently showed no significant effects on the parental and child`s psychosocial outcomes in our sample, except for child age among toddlers.

The study shows several strengths and limitations. To our knowledge, the CoronabaBY study is the first and only longitudinal study on psychosocial stress factors during the COVID-19 pandemic in families with children under three years. It covers an extensive period of the pandemic and considers a large sample in the follow-up evaluation despite the omission of dropouts. The questionnaires used are validated and established instruments for the assessment of parents’ and child's psychosocial stress. The study team developed the questions on pandemic-related burdens since appropriate questionnaires were not available. However, according to previous publications on the CoronabaBY study, significant correlations of the pandemic-related questions among each other and with other validated stress instruments (e.g., EBI) were evident (7, 8). Since all questionnaires had to be filled out completely before submission, there are no missing values, except for only a few cases where incorrect entries were made (e.g., parental age). Looking at the limitations, it has to be mentioned that mainly well-off, higher educated German mothers participated in the study. This is not uncommon, as is often the case in scientific studies (71), but could limit generalizability. Likewise, only parents using the app could participate. As all eligible families were invited—providing a quick and low-threshold access to the study due to high pandemic loads—this is a convenient sample. Furthermore, it was recruited in Bavaria and might not completely represent the German population. A further limitation is the high dropout rate of about two thirds. The dropouts showed significantly higher psychosocial stress already at baseline (i.e., parenting stress and parental affective symptoms). In addition, they exhibited significant differences in sociodemographic factors compared to the participants of the later follow-up (sample), indicating greater loads (e.g., being a single parent or having a child with a chronic illness and/or disability). As for the longitudinal perspective we merely compared participants with data at baseline and follow-up, further statements about the dropouts cannot be made. However, it has to be assumed that the psychosocial stress scores would have developed even more if these parents had also participated in the second part of the survey and could have been included in the follow-up. Thus, our results may have been underestimated.

In summary, a long-term trend can be identified over the pandemic with a mean interval of almost 40 weeks between baseline and follow-up. Although the CoronabaBY study started at the beginning of the second pandemic year, assuming that the highest loads were in the first year of COVID-19, intra-individual psychosocial stress factors in young families partly remained constantly high (crying/feeding/sleeping problems) or even increased (parenting stress) from the second into the third pandemic year. Hence, longitudinal effects of the pandemic on psychosocial health in these families seem to be present, although the pandemic and related burdens were fading through 2022. Our results indicate a need for action and can serve as a basis for decision-makers to better understand young families’ needs in times of crises and adapt or develop appropriate low-threshold support services for this vulnerable target group. Accessible support for parents and their children is indispensable, ensuring a healthy development of children as this goes along with parental mental health and well-being (72–77). Guidance can relieve parents psychologically by showing them resolutions for solving conflict situations and improving the family climate. Furthermore, ways can be identified to reduce parenting stress and thus strengthen the important ability to be emotionally available to the child's needs and ensuring appropriate care for the youngest. According to the present longitudinal results, existing measures and services seem to be insufficient to adequately support parents. This is particularly relevant as young families are facing further crises (e.g., armed conflicts, inflation, societal disparities, and climate change). Given these challenges, psychosocial stress factors in this target group might more and more increase and should therefore be further monitored and evaluated. This is the aim of the continuing JuFaBY study, which follows the CoronabaBY study and started in February 2023.

## Data Availability

The original contributions presented in the study are included in the article/supplementary material, further inquiries can be directed to the corresponding author, Catherine Buechel, catherine.buechel@tum.de.
